# Explosive strength: effect of knee-joint angle on functional, neural, and intrinsic contractile properties

**DOI:** 10.1007/s00421-019-04163-0

**Published:** 2019-05-21

**Authors:** Marcel Bahia Lanza, T. G. Balshaw, J. P. Folland

**Affiliations:** 1 School of Sport, Exercise, and Health Sciences, Loughborough University, Leicestershire, LE11 3TU UK; 2 CAPES Foundation, Ministry of Education of Brazil, Brasilia, DF 70040-020 Brazil

**Keywords:** Explosive torque, Neuromuscular activation, Muscle contractile properties

## Abstract

**Purpose:**

The present study compared knee extension explosive isometric torque, neuromuscular activation, and intrinsic contractile properties at five different knee-joint angles (35°, 50°, 65°, 80°, and 95°; 0° = full knee extension).

**Methods:**

Twenty-eight young healthy males performed two experimental sessions each involving: 2 maximum, and 6–8 explosive voluntary contractions at each angle; to measure maximum voluntary torque (MVT), explosive voluntary torque (EVT; 50–150 ms after contraction onset) and quadriceps surface EMG (QEMG, 0–50, 0–100, and 0–150 ms after EMG onset during the explosive contractions). Maximum twitch and M-wave (*M*_MAX_) responses as well as octet contractions were evoked with femoral nerve stimulation at each angle.

**Results:**

Absolute MVT and EVT showed an inverted ‘U’ relationship with higher torque at intermediate angles. There were no differences between knee-joint angles for relative EVT (%MVT) during the early phase (≤ 75 ms) of contraction and only subtle differences during the late phase (≥ 75 ms) of contraction (≤ 11%). Neuromuscular activation during explosive contractions was greater at more flexed than extended positions, and this was also the case during MVT. Whilst relative twitch torque (%MVT) was higher at knee flexed positions (*P* ≤ 0.001), relative octet torque (%MVT) was higher at knee extended positions (*P* ≤ 0.001).

**Conclusion:**

Relative EVT was broadly similar between joint angles, likely because neuromuscular activation during both explosive and plateau (maximum) phases of contraction changed proportionally, and due to the opposing changes in twitch and octet evoked responses with joint angle.

## Introduction

Explosive strength can be defined as the ability to increase contractile torque as quickly as possible from a low/resting level (Folland et al. 2014; Balshaw et al. 2016). Generating torque quickly is intuitively and empirically important for explosive athletic performance (Paasuke et al. 2001; Tillin et al. 2013a), but also appears to be critical to recovering from a loss of balance and thus preventing a fall (Izquierdo et al. 1999; Pijnappels et al. 2008; Behan et al. 2018). Whilst knee-joint maximum strength [i.e., maximum voluntary torque (MVT)] is known to be angle dependent (Kulig et al. 1984; Suter and Herzog 1997; Becker and Awiszus 2001; Newman et al. 2003), relatively little is known about the effect of joint angle on explosive strength and the underpinning neural and intrinsic contractile properties.

There are mixed reports for the effect of joint angle on absolute explosive voluntary torque (EVT, i.e., Nm) of the knee extensors. For instance, absolute EVT has been reported to be angle dependent throughout the rising phase of contraction with a similar angle–torque relationship as for MVT (Rousanoglou et al. 2010), and reported to be angle-independent during the early (≤ 75 ms; de Ruiter et al. 2004) and late phases (≥ 75 ms; Tillin et al. 2012) of contraction. Furthermore, EVT is often expressed relative to MVT, which reveals the ability to explosively express the available torque (i.e., MVT). The previous studies have documented relative early phase EVT to be: similar (de Ruiter et al. 2004; Tillin et al. 2012); or higher at extended vs. flexed knee-joint angles (Rousanoglou et al. 2010). The different results between previous studies may be due to the limited number of joint angles (2 angles, Tillin et al. 2012; 3 angles, de Ruiter et al. 2004), or EVT being measured only during the first 40 ms of EVC (de Ruiter et al. 2004). Overall, these inconsistent findings in the existing research literature make it difficult to discern the effect of joint angle on absolute and relative EVT.

EVT appears to be heavily influenced by neuromuscular activation (de Ruiter et al. 2004; Klass et al. 2008; Folland et al. 2014), which can be assessed with surface electromyography (EMG). Whilst it has previously been found that maximum quadriceps neuromuscular activation (i.e., at MVT) was lower at an extended vs. flexed knee-joint angles (Lanza et al. 2017), it is unclear if this is also the case during explosive contractions. One study reported higher activation during explosive contractions at a flexed vs. extended position; however, this study compared only two joint angles (Tillin et al. 2012). Hence, a more thorough investigation of multiple joint angles using contemporary EMG methods, such as the use of joint-angle specific *M*_MAX_ normalisation (e.g.  by normalising the EMG at each joint angle by *M*_MAX_ at that angle; Tillin et al. 2012; Lanza et al. 2017, 2018) and two EMG sensors per individual quadriceps muscle (Balshaw et al. 2017), seems warranted.

The intrinsic contractile properties of the muscle, specifically evoked torque during evoked twitch and octet [which drives the muscle at its maximal capacity for explosive torque production (de Haan 1998)] contractions, are also known to be determinants of EVT (Folland et al. 2014). The influence of joint angle on these contractile determinants of explosive strength has produced contrasting findings. For example, the contractile ability to rapidly express the available torque during evoked contractions (i.e., octet torque in proportion to MVT) has been reported to be dependent (i.e., higher at extended positions; Tillin et al. 2012) and independent (de Ruiter et al. 2004) of joint angle. Thus, little is known regarding how intrinsic contractile properties change across knee-joint angles.

Therefore, the purpose of this study was to conduct a thorough comparison of explosive torque production at five different knee-joint angles, with assessment of the underpinning physiological mechanisms, neuromuscular activation [via electromyography (EMG)], and intrinsic contractile properties, that might account for any differences between angles.

## Methods

### Participants

Twenty-eight, recreationally active males (age: 23 ± 3 years; height: 1.78 ± 0.07 m; body mass: 74 ± 7 kg) who were asymptomatic and had no history of major traumatic lower body injuries or participation in lower body strength/power training for ≥ 12 months participated in this study. The study was approved by the Loughborough University ethics committee. All participants provided written informed consent prior to their participation according to the principles of The Declaration of Helsinki.

### Overview

Participants attended three laboratory sessions (one familiarisation and two measurement sessions); each separated by 3–7 days, at a consistent time of the day and were instructed to avoid strenuous exercise in the 48 h prior to each session and any exercise in the previous 24 h. The first laboratory session was used to familiarise participants with voluntary and evoked unilateral isometric knee extension contractions of the dominant leg (by asking which leg the participant would kick a ball with) and involved identical procedures as the main sessions. Both measurement sessions involved a series of isometric knee extension contractions performed at five different knee-joint angles [35°, 50°, 65°, 80°, and 95° (0° = anatomical position), in a counter-balanced order (ascending or descending order in each session) and with a constant hip-joint angle of 65° (0° = anatomical position)]. Specifically, maximum voluntary contractions (MVCs); EVCs; and evoked twitch and octet contractions (8 pulses at 300 Hz, which were performed only in the second measurement session). Torque and superficial quadriceps femoris EMG [mean of vastus lateralis (VL), vastus medialis (VM) and rectus femoris (RF)], a measure of neuromuscular activation, were recorded during all tasks during both measurement sessions. In addition, the voluntary *T*_50_/octet *T*_50_ ratio was used as an additional measure of voluntary neural efficacy during the first 50 ms of contraction.

### Recording procedures

#### Torque, surface EMG, and video recording

All tasks were performed with participants seated on an adjustable rigid custom-made isometric knee extension dynamometer  (testing chair) and strapped across the chest and across the waist to minimise extraneous movement. To measure the knee-joint angles (35°, 50°, 65°, 80°, and 95°) sagittal plane video images of the leg were recorded during familiarisation using a video camera placed lateral to the participant (Panasonic HC-V110, Secaucus, New Jersey, US) during MVCs, specifically the angle between visible markers placed on the greater trochanter, lateral knee-joint space, and lateral malleolus. Video images were digitised using the freely available public domain analysis software (Kinovea 0.8.15 software).

Force was measured with a calibrated S-beam strain gauge (linear range 0–1500 N, Force Logic, Swallowfield, UK) which was attached perpendicular to the tibia with a reinforced inextensible webbing strap (35 mm width) fastened ~ 3 cm superior to the lateral malleolus. Force was sampled and recorded at 2000 Hz using an analogue-to-digital converter (A/D Micro 1401, CED, Cambridge, UK) and a computer utilising the Spike 2 software (CED, Cambridge, UK). A notch filter at 50 Hz with an infinite impulse response digital filter (*q*-factor of 10) was used to remove mains frequency noise and also a 500 Hz fourth-order zero-lag Butterworth digital low-pass filter was applied. Torque was calculated as the product of force and lever arm length (the distance between the knee-joint centre and the middle of the strap).

Surface EMG was recorded using a wireless EMG system (Trigno; Delsys Inc., Boston, MA). After preparing the skin by shaving, abrading, and cleansing with 70% ethanol, Trigno single differential sensors with a fixed 1-cm inter-electrode distance (Delsys Inc., Boston, MA) were attached over the superficial quadriceps muscles using adhesive interfaces. Six-independent EMG sensors (i.e., two per superficial constituent muscle; Balshaw et al. 2017) were located at the following percentages of thigh length (the distance from the knee-joint centre to the greater trochanter) above the superior border of the patella over the VM (25 and 35%), VL (50 and 60%), and RF (55 and 65%). EMG signals were amplified and filtered at source (× 300; 20–450-Hz bandwidth) before further amplification (overall effective gain, × 909) and subsequently sampled at 2000 Hz using the same external A/D converter and computer software as the force recordings. During the offline analysis, the EMG data were time aligned with the force signal by removing the inherent 48-ms delay of the Trigno EMG system.

### Protocol

#### Maximal voluntary contractions

Following a series of sub-maximum warm-up contractions at the first measured joint angle [50% (x2), 75% (x2), and 90% (x1) of perceived maximum effort], participants completed two MVCs at each of the five knee-joint angles. Participants were instructed to extend their knee and “push as hard as possible” for ~ 4 s during MVCs, with ≥ 30 s recovery between the pair of MVCs at each angle, but with at least 3 min recovery between successive joint angles. Biofeedback of the torque–time curve was displayed on a computer monitor in front of the participant during MVCs and a horizontal cursor was placed at the peak of the torque–time curve following the first MVC to encourage participants to exceed their best score. Intense verbal encouragement was also offered during all MVCs. During offline analysis, maximum voluntary torque (MVT) was identified as the instantaneous highest torque during both MVCs, and EMG amplitude of each individual EMG sensor at MVT (EMG_MVT_) was measured as the root mean square (RMS) during a 500 ms time window (250 ms either side of MVT). EMG_MVT_ was subsequently normalised to M_MAX_ peak-to-peak amplitude (*M*_MAX_ P–P; see below) recorded from the corresponding EMG sensor during maximum twitch contractions at the same angle (Lanza et al. 2018). The values from all 6 EMG sites were averaged to calculate a whole quadriceps value (QEMG_MVT_).

#### Explosive voluntary contractions

**Fig. 1:**
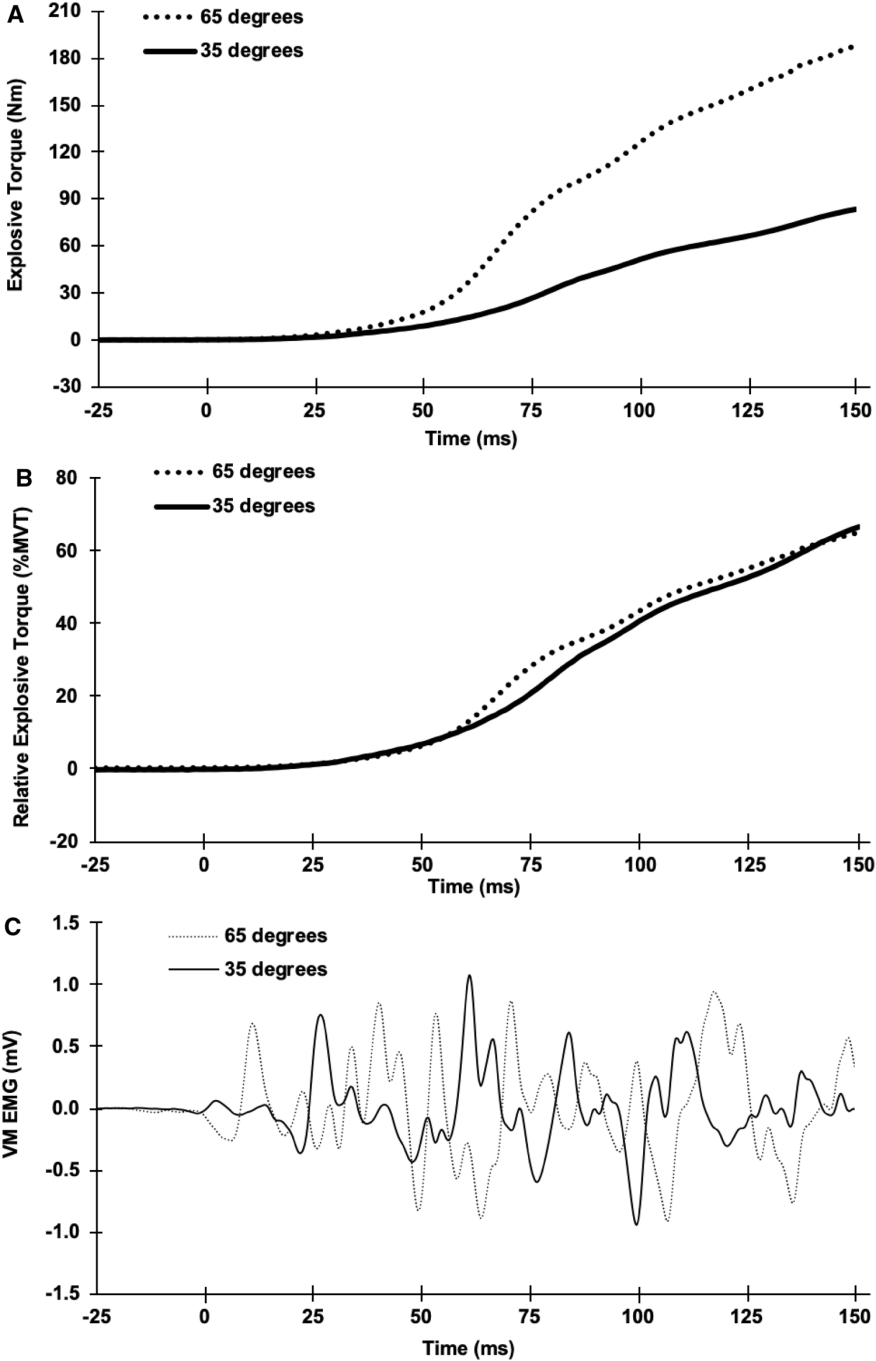
Example recordings from one participant during an explosive voluntary contractions at 35° and 65° knee-joint angles: **a** explosive torque and **b** explosive torque relative to maximum voluntary torque (%MVT) from torque onset (0 ms); **c** raw vastus medialis (VM) electromyography amplitude from EMG onset (0 ms)

After completing MVCs at all angles participants performed 6–8 EVCs (each ~ 20 s apart) at each knee-joint angle. Prior to each contraction, participants were instructed to perform the contraction as “fast and hard” as possible without producing prior tension or countermovement. Baseline torque was displayed on a sensitive *y*-axis scale (e.g., torque range of 0.61 Nm) was used to verify that pre-tension or countermovement did not occur before contraction onset and if either of these criteria were violated the contraction was excluded. Torque and EMG onsets for EVCs and evoked contractions (both twitch and octet; see below) were identified manually by visual identification by a trained investigator using a systematic approach, as previously described (Tillin et al. 2013a). Shortly, torque and EMG signals were viewed on an *x*-axis scale of 300 ms prior to the contraction and *y*-axis scales of 0.64 N m (torque) or 0.05 mV (EMG) (Tillin et al. 2013b; Balshaw et al. 2016). During offline analysis, for each knee-joint angle and measurement session, the two EVCs with the highest torque at 100 ms and peak force > 70% MVT were selected for further analysis. Explosive torque (an example of torque recording is shown in Fig. 1a) was measured at 50, 75, 100, and 150 ms from torque onset (*T*_50_, *T*_75_, *T*_100,_ and *T*_150,_ respectively) for each contraction, and then, a mean was calculated for the two contractions at each angle during each session, before averaging across measurement sessions. These timepoints (*T*_50_, *T*_75_, *T*_100,_ and *T*_150_) during explosive contractions were selected, as they typically represent consistent torque increments during the rising torque–time trace (i.e., ~ 20, 40, 60, and 80% MVT). Absolute explosive torque was also expressed relative to MVT (see Fig. 1b for an example) at the same timepoints to assess the capacity to use the available torque at each joint angle. The EMG amplitude (see Fig. 1c for an example) of each individual sensor during explosive contractions was measured across epochs of 0–50 (QEMG_0–50_), 0–100 (QEMG_0–100_), and 0–150 ms (QEMG_0–150_) from EMG onset and normalised to *M*_MAX_ P–P from the corresponding sensor measured during twitch contractions at the same knee-joint angle.

#### Evoked twitch and octet contractions

After the EVCs (i.e., at all 5 angles) and a 2 min rest, transcutaneous femoral nerve stimulation commenced. An anode (70 × 100 mm carbon rubber electrode; Electro-Medical Supplies, Greenham, UK) was placed and secured over the greater trochanter and a cathode (10 mm diameter, protruding 20 mm from a 35 × 55 mm plastic base; Electro-Medical Supplies, Greenham, UK) was positioned over the femoral nerve in the femoral triangle, with both coated in conductive gel. Electrical stimulation was then delivered with a constant-current variable-voltage stimulator (DS7AH, Digitimer Ltd., Welwyn Garden City, UK). The cathode was repositioned and a low-level current (40–60 mA) was delivered until the largest twitch torque response was identified. The cathode was secured with transpore tape and stimulation intensity (current) was gradually increased until torque and the peak-to-peak amplitude of the M-wave plateaued. Thereafter, three further stimuli, with 10 s inbetween each stimuli, were delivered with a current of 150% of the plateau level to ensure supramaximal M-wave (*M*_MAX_) peak-to-peak amplitude responses. Then, the angle was adjusted and the final stages of the current calibration and three supramaximal stimuli were repeated at each new angle (ascending/descending order of angles within the two measurement sessions). Twitch peak torque (twitch PT), twitch torque at 50 ms (twitch *T*_50_), and *M*_MAX_ P–P were averaged across the three supramaximal evoked contractions at each joint angle.

Evoked octet contractions were performed during the second (final) measurement session only, after all other procedures had been completed at all joint angles. Octets were first evoked at progressive currents (~ 15 s apart) until a plateau in the amplitude of peak torque and peak rate of torque development were achieved (only at the first angle tested). Then, two discrete pulse trains (≥ 15 s apart) were delivered with a higher current (≥ 20% above the plateau current to ensure supramaximal stimulation) to evoke maximum octet contractions. Octets were performed in a counter-balanced order (i.e., half of the participants performed the octets in the order most flexed to most extended and the other half in the opposite order). Octet peak torque (octet PT) and octet torque at 50 ms (octet *T*_50_) were measured as the mean across the two maximum evoked octet contractions at each angle. The ratio of voluntary *T*_50_/octet *T*_50_ at each joint angle was used as an additional measure of volitional neural efficacy during the EVCs. Absolute twitch and octet torque variables were also expressed relative to MVT. Due to the discomfort caused by octet stimulation, two participants were unable to tolerate this measurement reducing the sample size to 26 participants for octet torque measurements.

### Data analysis and statistics

Data are reported as mean ± standard deviation of the mean (SD) and all variables (except octet measures) were averaged between the two measurement sessions before any statistical analysis was conducted. Between-session reliability of MVT, EVT, *M*_MAX_ P–P (whole Q), QEMG (at MVT and during EVCs), and twitch torque variables from the main trial sessions were assessed by calculating within-participant coefficient of variation [CV_W_; (SD/mean) ×v100]. CV_W_ values presented are averaged across the five knee-joint angles. A one-way repeated measures general linear model ANOVA was used to determine if there were differences between knee-joint angles for each variable. When the ANOVA revealed differences between the five angles, Bonferroni post-hoc test with corrections was used to perform pairwise comparisons between angles. To identify if the optimal angle for voluntary torque production was influenced by the time/phase of contraction individual torque–angle relationships for each measure (EVT at each timepoint and MVT) were fitted with a second order polynomial function and the optimal angle derived. Optimum angles for the group were compared between EVT timepoints and MVT. Statistical analysis was performed using SPSS version 23 (IBM Corporation, Armonk, New York, USA), and the significance level was set at *P* < 0.05.

## Results

### Reliability

MVT had a between-session CV_W_ of 5.4% (collapsed across all five angles) followed by *T*_75_, *T*_150,_ and *T*_100_ (CV_W_ of 7.6%, 7.8%, and 10.6%, respectively) and finally *T*_50_ with the highest CV_W_ (24.4%). Evoked twitch *T*_50_ (8.5%) and twitch PT (9.0%) showed a similar CV_W_ and *M*_MAX_ P–P had a CV_W_ of 7.5%. Normalised EMG variables displayed a CV_W_ of 14.6% for QEMG_MVT_, 19.8% for QEMG_0–50_, 16.0% for QEMG_0–100,_ and 14.2% for QEMG_0–150_.

### Voluntary torque

#### Absolute torque

**Fig. 2:**
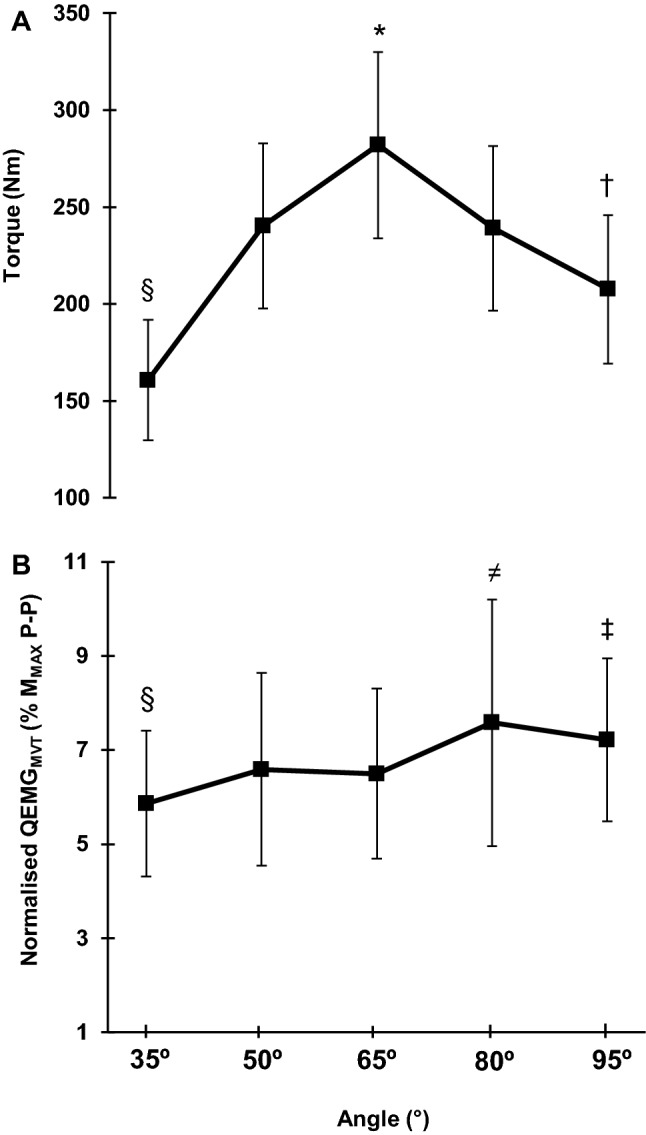
Knee extensor maximum voluntary torque (MVT)– and QEMG_MVT_–angle relationships during maximum isometric contractions performed at knee-joint angles of 35°, 50°, 65°, 80°, and 95°. Both relationships, MVT–angle (**a**) and QEMG_MVT_–angle (**b**), showed a main effect of angle (one-way ANOVA P < 0.001) with post-hoc differences denoted by: *higher than the other angles, ^§^lower than the other angles, ^†^lower than 50° and 80°, ^**≠**^higher than 35°, 50°, and 65°, and ^‡^higher than 65°. Data are mean ± SD (*n *= 28). *M*_*MAX*_*P–P* supramaximal M-wave peak-to-peak amplitude, *QEMG*_*MVT*_ quadriceps femoris EMG at maximum voluntary torque

**Fig. 3:**
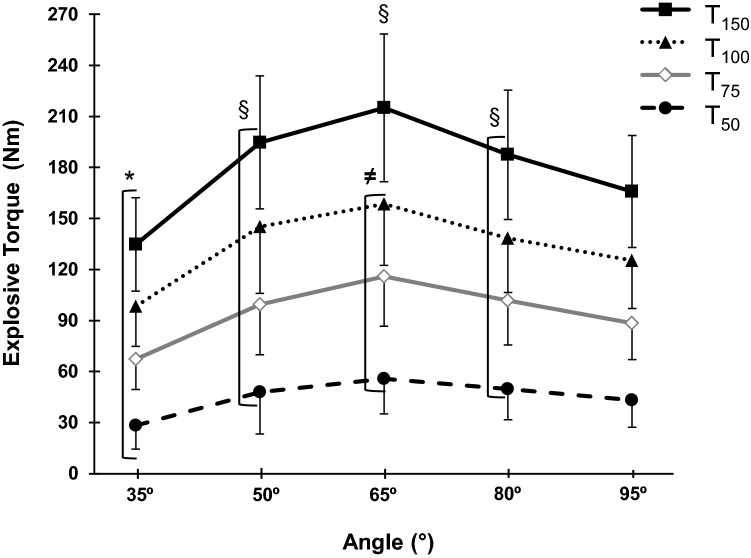
Knee extensor absolute explosive torque–angle relationship measured at four timepoints (50, 75, 100, and 150 ms) after torque onset during explosive voluntary contractions at knee-joint angles of 35°, 50°, 65°, 80°, and 95°. One-way ANOVA showed an effect of angle for all timepoints (*P* < 0.001), with post-hoc differences denoted by: *lower than the other angles; ^§^higher than 95°;  and ^≠^higher than the other angles (in relation to all bracketed timepoints). Data are mean ± SD (*n *= 28)

As expected, the MVT–angle relationship displayed a distinct ‘inverted-U’ relationship (Fig. 2a), and explosive torque at all timepoints showed a similar pattern/relationship with joint angle (one-way ANOVA, *P* < 0.001; Table 1). Post-hoc analysis revealed that for MVT, *T*_75_, *T*_100,_ and *T*_150,_ there was higher torque at 65° than all other angles (*P* < 0.001); lower torque at 35° (up to 42% lower) than all other positions (*P* < 0.001); and torque at 50° and 80° was higher than 95° (up to 20% higher; *P* ≤ 0.040; Fig. 3). Differences in torque after 50 ms of explosive contraction were slightly less pronounced, with 35° displaying up to 49% lower values than the other positions (*P* < 0.001) and 95° being 22% lower than 65° and 14% lower than 80° (*P* ≤ 0.003; Fig. 3). The optimal angle for voluntary torque production, based on the quadratic fit of individual angle–torque relationships, was unaffected by time/phase of contraction (EVT at each timepoint and MVT).

**Table 1:** Absolute intrinsic contractile properties and supramaximal compound muscle action potential peak-to-peak amplitude (*M*_MAX_ P–P) assessed by evoked contractions at knee-joint angles of 35°, 50°, 65°, 80°, and 95° (where 0° = full extension)

	Knee-joint angle					One-way ANOVA (*P* value)
	35°	50°	65°	80°	95°	
Evoked torque (Nm)						
Twitch *T*_50_^¥^	24 ± 7	41 ± 10	52 ± 13	48 ± 11	46 ± 10	< 0.001
Twitch PT^¥^	30 ± 8	48 ± 10	59 ± 13	56 ± 13	53 ± 12	< 0.001
Octet *T*_50_^∞^	81 ± 15	120 ± 20	128 ± 23	100 ± 18	86 ± 16	< 0.001
Octet PT^‡^	129 ± 21	188 ± 30	199 ± 30	164 ± 34	146 ± 29	< 0.001
Evoked M-wave (mV)						
*M*_MAX_ P–P^¥^	4.0 ± 1.3	3.8 ± 1.3	3.6 ± 1.2	3.5 ± 1.1	3.3 ± 1.1	< 0.001

Data are mean ± SD (*n *= 28 for twitch torque and m-wave; *n *= 26 for octet torque). *PT* peak torque, *T*_*50*_ torque at 50 ms. When one-way ANOVAs detected a main effect for angle, post-hoc test (Bonferroni with correction) was used to determine the differences across angles. Symbols indicate: ^¥^differences between all knee-joint angles, ^∞^differences between all knee-joint angles except for 35° and 95° which were similar, and ^‡^differences between all knee-joint angles except for 50° and 80° which were similar

#### Relative torque

**Fig. 4:**
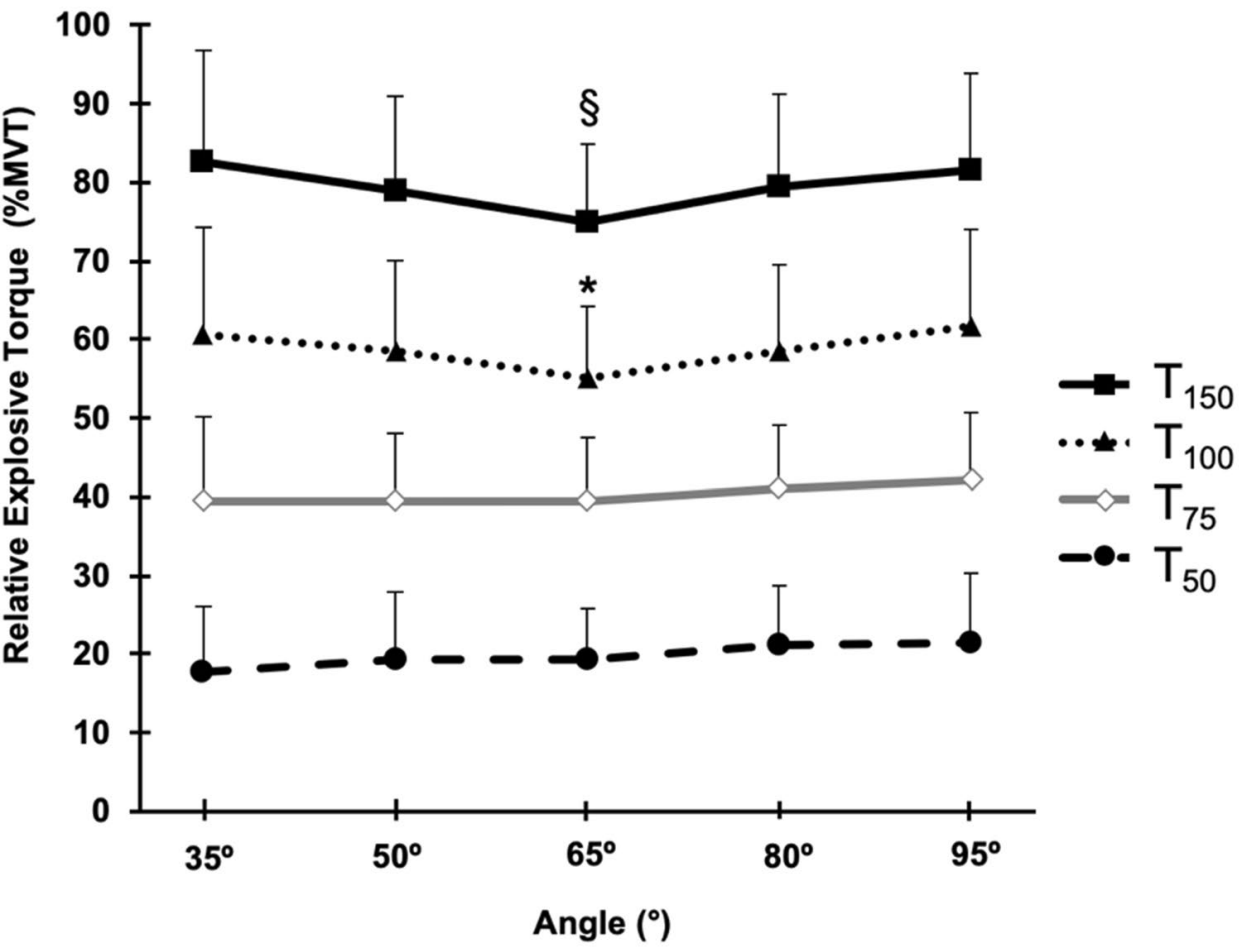
Knee extensor relative explosive torque [% maximum voluntary torque (MVT)]-angle relationship measured at four timepoints (50, 75, 100 and 150 ms) after torque onset during explosive voluntary contractions at knee-joint angles of 35°, 50°, 65°, 80°, and 95°. One-way ANOVA showed an effect of angle for *T*_100_ and *T*_150_ only (*P* ≤ 0.025) with post-hoc differences denoted by: ^*^lower than 80° and 95°; ^§^lower than the other angles. Data are mean ± SD (*n *= 28)

EVT expressed as a percentage of MVT presented a main effect of angle during the late phase of contraction (*T*_100_ and *T*_150_; one-way ANOVA, *P* ≤ 0.035; Fig. 4), but there was no effect of angle during the early phase of contraction (*T*_50_ and *T*_75_; one-way ANOVA, *P* ≥ 0.058; Fig. 4). Subsequent post-hoc tests for the late phase of contraction showed relatively modest differences in relative EVT: *T*_100_ at 65° was lower than at 80° (5.8%) and 95° (10.6%; *P* ≤ 0.019); *T*_150_ was lower at 65° than all the other angles (5.0–9.3%; *P* ≤ 0.041; Fig. 4).

### Neuromuscular activation

**Fig. 5:**
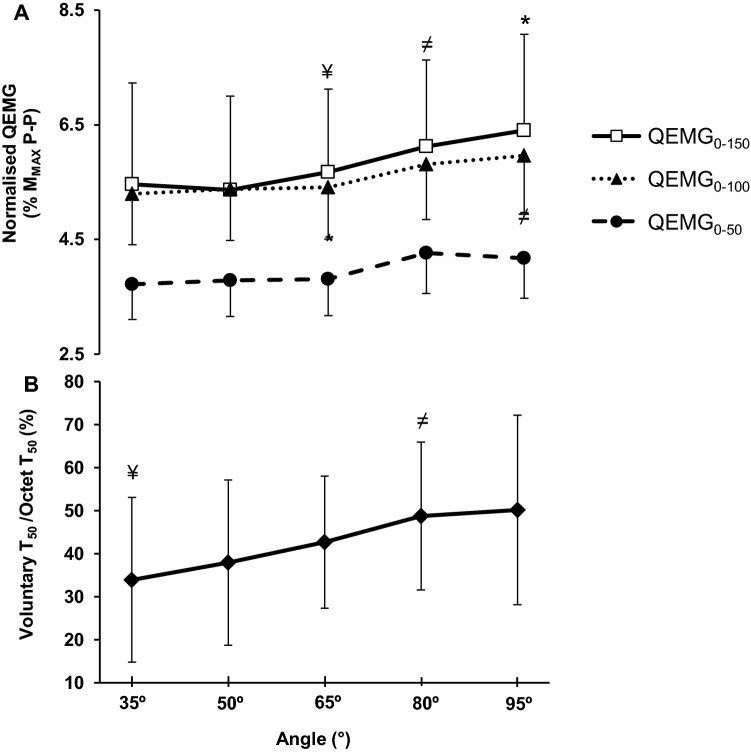
**a** Normalised QEMG–angle relationships at 0–50, 0–100, and 0–150 ms during explosive voluntary contraction (EVC) and **b***T*_50_ relative to octet *T*_50_ at knee-joint angles of 35°, 50°, 65°, 80°, and 95°. One-way ANOVA showed differences between angles for QEMG_0–100_ and QEMG_0–150_ (*P* ≤ 0.013). One-way ANOVA showed differences between angles for voluntary explosive contraction at *T*_50_ relative to octet *T*_50_ (*P* < 0.001). Symbols should be considered at the top of the error bars and indicate: ^¥^lower than 80° and 95°, ^*^higher than the others, ^≠^higher than 50°. Data are mean ± SD (*n *= 28 for EMG; *n *= 26 for voluntary *T*_50_/octet *T*_50_ ratio). *T*_*50*_ torque at 50 ms after contraction onset, *M*_*MAX*_*P–P* supramaximal M-wave peak-to-peak amplitude

Normalised QEMG_MVT_ (%*M*_MAX_ P–P) presented a main effect for angle (one-way ANOVA, *P* = 0.001) and post-hoc tests revealed: lower activation at the most extended position (35°; − 7 to − 19%) compared to more flexed angles (65°, 80°, 95° *P* ≤ 0.001); 50° (− 16%) and 65° (− 13%) was lower than 80°, respectively (*P* ≤ 0.028; Fig. 2b). During EVC, normalised QEMG_0–100_ and QEMG_0–150_ displayed a main effect for angle (one-way ANOVA, both *P* < 0.001; Fig. 5a), but normalised QEMG_0–50_ showed no main effect of angle (one-way ANOVA, *P* = 0.064; Fig. 5a). Normalised QEMG_0–100_ was lower at more extended vs. flexed knee-joint angles, with 50° and 65° < 95° (*P* ≤ 0.022), whilst only 65° < 80° (*P* = 0.040; Fig. 5a). Similarly, normalised QEMG_0-150_ was also lower at more extended than flexed knee-joint positions with 35°, 50° and 65° < 95° (*P* ≤ 0.004), and 50° and 65° < 80° (*P* ≤ 0.013; Fig. 4a).

When voluntary explosive *T*_50_ was expressed relative to evoked octet *T*_50_, indicative of neural efficacy, it showed a main effect of joint angle (one-way ANOVA, *P* ≤ 0.001; Fig. 5b) with post-hoc differences demonstrating: 35° < 80° and 95° (*P* ≤ 0.013); and 50° < 80° (*P* = 0.018), Fig. 5b.

#### M_MAX_ peak-to-peak

*M*_MAX_ P–P presented a main effect of angle (one-way ANOVA; Table 1) with the highest values at the most extended position, all angles being different from each other, and progressively (lower) at increasingly flexed positions (Bonferroni post-hoc, *P* ≤ 0.001).

### Evoked responses and intrinsic contractile properties

#### Absolute involuntary torque

Absolute torque during evoked contractions (twitch *T*_50_, twitch PT, octet *T*_50,_ and octet PT) showed a main effect for angle (one-way ANOVA; Table 1). For evoked twitch *T*_50_, twitch PT, and octet PT all angles were different from each other (*P* ≤ 0.048; Table 1) with the shape of the torque–angle relationship similar to MVT (i.e., an inverted ‘U’). Differences between angles for evoked octet *T*_50_ were found for almost all angles (all *P* < 0.001), the exception was the extreme angles which were similar (35° and 95°; *P* = 0.735; Table 1).

#### Relative involuntary torque

**Fig. 6:**
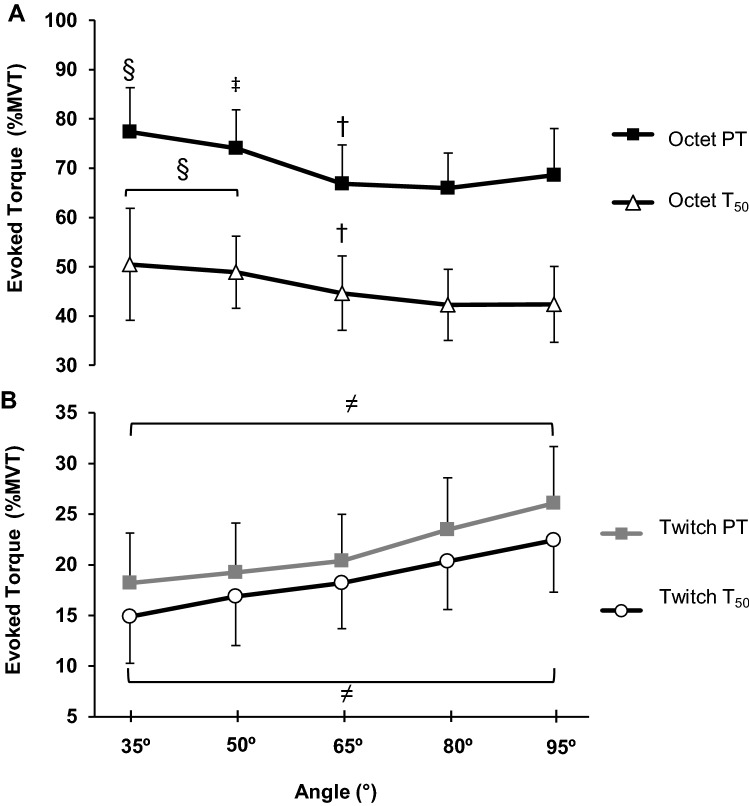
Evoked knee extensor relative torque (relative to MVT)-angle relationship for **a** evoked octet and **b** twitch contractions at knee-joint angles of 35°, 50°, 65°, 80°, and 95°. There was a main effect of angle for twitch and octet *T*_50_ (torque at 50 ms after onset) and also twitch and octet PT (peak torque; *P* < 0.001). Symbols indicate: ^≠^differences between all knee-joint angles, ^§^higher than 65°, 80°, and 95°, ^‡^higher than 65° and 80°, and ^†^higher than 80°. Data are mean ± SD (*n *= 28 for twitch torque; *n *= 26 for octet torque)

When evoked torque variables were expressed relative to MVT, a main effect of angle was detected (one-way ANOVA *P* < 0.001; Fig. 6) and post-hoc tests showed all angles were different from each other for all evoked variables (*P* < 0.001). However, relative twitch torques (*T*_50_ and PT) increased with knee-joint angle (i.e., up to 34% greater at flexed vs. extended positions; Fig. 6a), whereas relative evoked octet torques (*T*_50_ and PT) decreased with knee-joint angle (i.e., up to 16% lower at flexed vs. extended positions; Fig. 6b).

## Discussion

The present study compared voluntary torque production and neuromuscular activation during explosive and maximum contractions, as well as the evoked contractile properties relevant to explosive voluntary torque, across knee-joint angles. As expected MVT was highly angle-dependent (Suter and Herzog 1997; Becker and Awiszus 2001; Newman et al. 2003) and absolute EVT largely followed the same torque–angle relationship as MVT. Consequently, the ability to explosively express the available torque, i.e., EVT relative to MVT, also showed no effect of joint angle during the early phase of contraction (≤ 75 ms) and only subtle differences (< 11%) during the later phase of contraction (> 75 ms). Neuromuscular activation during explosive contractions increased with knee flexion angle, which was also the case during MVCs, and likely explains why relative explosive torque was broadly consistent across angles as activation during both types of contractions changed in a similar manner. In addition, the contractile response to evoked twitch and octet contractions showed opposite effects of joint angle, increasing and decreasing with knee flexion, respectively.

As expected absolute EVT at all timepoints during the rising phase of contraction was angle-dependent in a similar manner to MVT, with the highest values at 65° and lower values at more flexed and extended knee-joint positions (Fig. 3). Whilst early phase EVT has previously been reported not to vary with knee-joint angle, these experiments used a limited number of joint angles that likely provides an incomplete picture of the explosive torque–angle relationship and substantially smaller numbers of participants than the current study (*n *= 7, 3 angles, de Ruiter et al. 2004; *n *= 14, 2 angles, Tillin et al. 2012). Considering the changes in cross-bridge overlap with muscle length and thus joint angle (i.e., optimal overlap at a mid-range length; Lieber et al. 1994; Rassier et al. 1999), it is to be expected that a mid-range joint angle would be optimum for both maximum and explosive torque production as we have found.

The ability to express the available torque generating capacity explosively (i.e., relative EVT) during the early phase of contraction was independent of knee-joint angle in the current study, although there were subtle differences in late phase relative explosive torque (100 and 150 ms) with surprisingly the middle angle (65°) being up to 11% lower than other angles. Hence, overall there was a relatively consistent ability to explosively express the available torque across knee-joint angles. In agreement with our findings, early phase relative EVT has been found to be similar for different knee-joint angles (de Ruiter et al. 2004; Tillin et al. 2012), although in contrast to our findings during the later phase of EVC, higher relative EVT has been reported at more extended knee-joint positions (Rousanoglou et al. 2010; Tillin et al. 2012). The presented findings may be explained by neuromuscular activation and muscle contractile properties.

Neuromuscular activation assessed with normalised QEMG was higher at flexed than extended positions during MVT (QEMG_MVT_ up to + 18%) and also during the first 100 and 150 (QEMG_0–100_ + 11%; QEMG_0–150_, + 16%), but not the first 50 ms (QEMG_0–50_), of explosive contractions. However, it is notable that QEMG_0–50_ was the least reliable EMG measure in this study (CV 19.8%), likely due to the short time period of this measurement. Furthermore, neural efficacy (voluntary/evoked octet *T*_50_ ratio) an additional indicator of volitional activation during the first 50 ms of EVC was higher at more flexed positions (up to 34%) in a similar manner to the EMG measures. Based on this data, neuromuscular activation was effected by joint angle throughout explosive contraction as well as at MVT, being greater at more flexed positions. These findings are consistent with our previous study that found lower activation, assessed with both normalised EMG and the interpolated twitch technique, during MVCs at extended vs. flexed knee-joint angles (Lanza et al. 2017). In combination both studies provide robust evidence that neuromuscular activation is sensitive to knee-joint angle during open kinetic chain tasks. There are some suggestions that closed kinetic chain tasks (i.e., leg press) may also have higher neuromuscular activation at more flexed knee positions (Escamilla et al. 1998; Walker et al. 2011). However, these studies have not involved such rigourous normalisation (i.e., to *M*_MAX_) and thus further investigation of closed kinetic chain activities with *M*_MAX_ normalisation would be informative. Moreover, as neuromuscular activation during the explosive (EVC) and plateau (MVC) phases of contraction both appear to be effected by joint angle in a similar manner, this may explain why relative explosive torque (i.e., expressed as a proportion of MVT) was broadly independent of joint angle.

The anterior displacement of the tibia has been suggested as an explanation for the changes in neuromuscular activation across joint angles during MVC (Suter and Herzog 1997; Lanza et al. 2017), and may also explain the changes in neuromuscular activation during the maximum and explosive contractions in the current study. Specifically, anterior displacement of the tibia at more extended positions (Hirokawa et al. 1992) may place greater stress on the anterior cruciate ligament (ACL) activating reflex pathways (i.e., Ia and Ib interneurons) that provide inhibitory input to the alpha motorneurones and thus limit neuromuscular activation (Johansson et al. 1991). Alternatively, it has been hypothesised that at longer muscle lengths (i.e., flexed positions) the muscle spindles are more active, producing higher Ia afferent excitatory input to the quadriceps motoneuron pool resulting in an increase in neuromuscular activation (Becker and Awiszus 2001; Kubo et al. 2004). In any case, the apparent effect of lower neuromuscular activation at extended knee-joint angles may moderate loading on the ACL and thus reduce injury risk. Furthermore, our findings may also have practical implications for our understanding of resistance training adaptations by indicating greater scope to increase neuromuscular activation with training at extended knee-joint angles. The angle/length dependence of *M*_MAX_ P–P (higher at short muscle lengths and extended angles) that we have seen in this and a previous experiment (Lanza et al. 2017) may be explained by the increase in muscle length leading to greater spatial distribution of the neuromuscular junctions and thus greater temporal distribution of the individual fibre action potentials and thus a more diffuse and smaller amplitude compound muscle action potential (*M*_max_).

A novel finding of the current study was that normalised evoked octet torque (*T*_50_ and PT, relative to MVT) was greatest in extended positions, whereas evoked twitch torque (*T*_50_ and PT, relative to MVT) was higher in more flexed positions (Fig. 6). Several animal studies have documented higher evoked twitch torque in a lengthened positions (Roszek et al. 1994; Rassier et al. 1998; Rassier and MacIntosh 2002) that has been attributed to increased Ca^2+^ sensitivity, which would be expected to increase the number of cross bridges formed, and so produce higher torque (Rassier and MacIntosh 2002). This mechanism seems a feasible explanation for the increased relative twitch torque we have observed at flexed knee-joint angles, where the quadriceps is lengthened.

Other animal work with tetanic stimulation found higher normalized evoked torque to occur at short muscle lengths (de Ruiter et al. 2008), and two human studies have also found higher evoked octet torque at short muscle lengths (de Ruiter et al. 2004; Debenham and Power 2018). It has been suggested that at longer muscle lengths, the heterogeneity of sarcomere lengths, with some sarcomeres shortening by significantly more than others, impairs relative explosive force/torque production. In contrast this phenomenon of heterogeneous sarcomere lengths is thought to be less pronounced, and  potentially favours, relative explosive force production at short lengths (de Ruiter et al. 2008). Therefore, the heterogeneity of sarcomere lengths might explain the evoked octet torque being higher at more flexed knee-joint angles (35° and 50°) and shorter muscle lengths than the optimum position (65°) and also more flexed positions. Furthermore, these opposite changes in the intrinsic contractile properties of evoked twitch vs. octet responses may provide an additional explanation for why relative voluntary explosive torque was largely independent of knee-joint angle.

The present study has some limitations and strengths that should be highlighted. A strength and a weakness of the study was the use of five different knee-joint angles that provided a number of data points for comparison between angles, but also involved quite a large number of contractions (10 MVCs and ~ 35 EVCs per measurement session). Whilst there were extensive rest periods built into the protocol and we do not consider the EVCs (contractions of < 1-s duration) to be particularly fatiguing, the current study did not have any objective assessment of fatigue to quantify if fatigue did occur. Nonetheless the current study used a counter-balanced design with an opposite angle order during the two measurement sessions to reduce the possibility of systematic order effects, including fatigue, learning or changes in attention. The current study also had a large number of participants to reduce the chance of random findings that can occur with small populations (Ludbrook 1995). One potential well-known limitation of sEMG measurements when comparing different angles is the difference in recording conditions between positions (Rainoldi et al. 2000). In this study, we used *M*_MAX_ normalisation to try and control for any changes in the electrical recording conditions and we also used two EMG electrodes over each of the superficial quadriceps muscles to get a more thorough assessment of quadriceps neuromuscular activation. To further understand the mechanisms behind the differences in neuromuscular activation between knee-joint angles, the use of high-density EMG decomposition and transcranial magnetic stimulation may provide further information and should be considered by future studies.

## Conclusion

To conclude, the absolute explosive torque–angle relationship exhibited the highest torques at mid-range knee-joint angles (65° from full extension) in a similar manner to MVT. Whilst the ability to explosively express the available torque (i.e., relative to MVT) revealed only subtle differences across joint angles during the later phase of contraction. Neuromuscular activation increased from extended to flexed positions during early and late phases of the explosive contractions as well as during MVCs. The similar changes in neuromuscular activation during explosive and maximum contractions may explain why relative explosive torque (%MVT) was broadly independent of joint angle. Finally, the opposing effects of joint angle on relative evoked twitch *T*_50_ and octet *T*_50_, increasing and decreasing with knee flexion angle, respectively, may have also contributed to the consistent relative voluntary explosive torque across knee-joint angles.

## Abbreviations

CV_W_: Within-participant coefficient of variation
EMG: Electromyography
EMG_MVT_: EMG at maximum voluntary torque
EVC: Explosive voluntary contraction
EVT: Explosive voluntary torque
*M*_MAX_: Supramaximal muscle compound action potential
*M*_MAX_ P–P: *M*_MAX_ peak-to-peak amplitude
MVC: Maximum voluntary contraction
MVT: Maximum voluntary torque
Octet PT: Octet peak torque
Octet *T*_50_: Octet torque measure at 50 ms after torque onset
QEMG_0–50_: Quadriceps femoris EMG epoch between 0 and 50 ms after EMG onset
QEMG_0–100_: Quadriceps femoris EMG epoch between 0 and 100 ms after EMG onset
QEMG_0–150_: Quadriceps femoris EMG epoch between 0 and 150 ms after EMG onset
QEMG_MVT_: Quadriceps femoris EMG at maximum voluntary torque
RF: Rectus femoris
RMS: Root mean square
*T*_50_: Explosive torque 50 ms after torque onset
*T*_75_: Explosive torque 75 ms after torque onset
*T*_100_: Explosive torque 100 ms after torque onset
*T*_150_: Explosive torque 150 ms after torque onset
Twitch PT: Twitch peak torque
Twitch *T*_50_: Twitch torque measured 50 ms after torque onset
VL: Vastus lateralis
VM: Vastus medialis
